# Supplementary material for: The Role of ABO Blood Type in Patients with SARS-CoV-2 Infection: A Systematic Review
Source: J Clin Med. 2022 May 27;11(11):3029. doi: 10.3390/jcm11113029 (PMC9181227; doi:10.3390/jcm11113029)
Supplement: Supplementary file 1 [file jcm-11-03029-s001.zip › jcm-1700023-supplementary.pdf]

ABO blood type & SARS-CoV-2_Search strategy

MEDLINE (Pubmed) (2020 to May 2021)

"COVID-19"[Mesh]) OR "SARS-CoV-2 variants" [Supplementary Concept]) OR "SARS-CoV-2"[Mesh]) OR “2019 novel coronavirus disease” OR COVID19 OR “COVID-19 pandemic” OR “SARS-CoV-2 infection” OR “COVID-19 virus disease” OR “2019 novel coronavirus infection” OR “2019-nCoV infection” OR “coronavirus disease 2019” OR “coronavirus disease-19” OR “2019-nCoV disease” OR “COVID-19 virus infection” OR “2019-nCoV” OR “Wuhan coronavirus” OR “SARS-CoV-2” OR “2019 novel coronavirus” OR “COVID-19 virus” OR “coronavirus disease 2019 virus” OR “COVID19 virus” OR “Wuhan seafood market pneumonia virus” AND "ABO Blood-Group System"[Mesh]) OR "Rh-Hr Blood-Group System"[Mesh]) OR ("ABO Blood Group System*"[Title/Abstract] OR "ABO Blood-Group System*"[Title/Abstract] OR "ABO Factor*"[Title/Abstract] OR "ABO blood group*"[Title/Abstract] OR "blood type"[Title/Abstract] OR "blood group*"[Title/Abstract] OR "ABO Group*"[Title/Abstract] OR "Blood type A"[Title/Abstract] OR "Blood type B"[Title/Abstract] OR "Blood type AB"[Title/Abstract] OR "Blood type O"[Title/Abstract] OR "ABO blood type*"[Title/Abstract] OR "ABO Blood-Group System*"[Title/Abstract] OR "Rh-Hr Blood-Group System"[Title/Abstract] OR "Rh Hr Blood Group System*"[Title/Abstract] OR "Rhesus Blood-Group System*"[Title/Abstract] OR "Rhesus Blood Group System*"[Title/Abstract]

Embase (2020 to May 2021)

#1 'covid 19'/exp
#2 '2019 novel coronavirus disease' OR covid19 OR 'covid-19 pandemic' OR 'sars-cov-2 infection' OR 'covid-19 virus disease' OR '2019 novel coronavirus infection' OR '2019-ncov infection' OR 'coronavirus disease 2019' OR 'coronavirus disease-19' OR '2019-ncov disease' OR 'covid-19 virus infection' OR '2019-ncov' OR 'wuhan coronavirus' OR 'sars-cov-2' OR '2019 novel coronavirus' OR 'covid-19 virus' OR 'coronavirus disease 2019 virus' OR 'covid19 virus' OR 'wuhan seafood market pneumonia virus' OR ncov OR covid:ti,ab
#5 #1 OR #2 OR #3 OR #4
#6 'blood group abo system'/exp/mj OR 'blood group rhesus system'/exp/mj
#7 'abo blood group* system*' OR 'abo factor*' OR 'abo blood group*' OR 'blood type' OR 'blood group*' OR 'abo group*' OR 'blood type a' OR 'blood type b' OR 'blood type ab' OR 'abo blood type*' OR 'abo blood-group system*' OR 'rh-hr blood-group system' OR 'rh hr blood group system*' OR 'rhesus blood-group system*' OR 'rhesus blood group system*' OR 'rh factor*' OR 'rh factor':ti,ab
#8 #6 OR #7
#9 #5 AND #8
#10 #5 AND #8 AND [embase]/limAND [2020-2021]/py

Cochrane Central Register of Controlled Trials (CENTRAL; 2021, Issue 5) in the Cochrane Library (searched May 2021)

#1 MeSH descriptor: [Coronavirus Infections] explode all trees
#2 MeSH descriptor: [COVID-19] explode all trees
#3 MeSH descriptor: [] explode all trees
#4 MeSH descriptor: [SARS-CoV-2] explode all trees
#5 ('Coronavirus Infection'):ti,ab,kw
#6 ('2019 novel coronavirus disease'):ti,ab,kw
#7 'COVID-19 pandemic'
#8 (COVID19):ti,ab,kw
#9 ('COVID-19 pandemic'):ti,ab,kw
#10 ('SARS-CoV-2 infection' OR 'COVID-19 virus disease'):ti,ab,kw
#11 '2019 novel coronavirus infection'
#12 ('coronavirus disease 2019'):ti,ab,kw
#13 ('coronavirus disease-19' OR 'COVID-19 virus infection'):ti,ab,kw

Table S1: ABO blood type & SARS-CoV-2_Search strategy

NEWCASTLE - OTTAWA QUALITY ASSESSMENT SCALE
CASE CONTROL STUDIES

Note: A study can be awarded a maximum of one star for each numbered item within the Selection and Exposure categories. A maximum of two stars can be given for Comparability.

Selection

1) Is the case definition adequate?
   a) yes, with independent validation **Ø**
   b) yes, eg record linkage or based on self reports
   c) no description

2) Representativeness of the cases
   a) consecutive or obviously representative series of cases **Ø**
   b) potential for selection biases or not stated

3) Selection of Controls
   a) community controls **Ø**
   b) hospital controls
   c) no description

4) Definition of Controls
   a) no history of disease (endpoint) **Ø**
   b) no description of source

Comparability

1) Comparability of cases and controls on the basis of the design or analysis
   a) study controls for _______________ (Select the most important factor.) **Ø**
   b) study controls for any additional factor **Ø** (This criteria could be modified to indicate specific control for a second important factor.)

Exposure

1) Ascertainment of exposure
   a) secure record (eg surgical records) **Ø**
   b) structured interview where blind to case/control status **Ø**
   c) interview not blinded to case/control status
   d) written self report or medical record only
   e) no description

2) Same method of ascertainment for cases and controls
   a) yes **Ø**
   b) no

3) Non-Response rate
   a) same rate for both groups **Ø**
   b) non respondents described
   c) rate different and no designation

NEWCASTLE - OTTAWA QUALITY ASSESSMENT SCALE
COHORT STUDIES

Note: A study can be awarded a maximum of one star for each numbered item within the Selection and Outcome categories. A maximum of two stars can be given for Comparability

Selection

1) Representativeness of the exposed cohort
   a) truly representative of the average _______________ (describe) in the community Ø
   b) somewhat representative of the average ______________ in the community Ø
   c) selected group of users eg nurses, volunteers
   d) no description of the derivation of the cohort

2) Selection of the non exposed cohort
   a) drawn from the same community as the exposed cohort Ø
   b) drawn from a different source
   c) no description of the derivation of the non exposed cohort

3) Ascertainment of exposure
   a) secure record (eg surgical records) Ø
   b) structured interview Ø
   c) written self report
   d) no description

4) Demonstration that outcome of interest was not present at start of study
   a) yes Ø
   b) no

Comparability

1) Comparability of cohorts on the basis of the design or analysis
   a) study controls for _____________ (select the most important factor) Ø
   b) study controls for any additional factor Ø (This criteria could be modified to indicate specific control for a second important factor.)

Outcome

1) Assessment of outcome
   a) independent blind assessment Ø
   b) record linkage Ø
   c) self report
   d) no description

2) Was follow-up long enough for outcomes to occur
   a) yes (select an adequate follow up period for outcome of interest) Ø
   b) no

3) Adequacy of follow up of cohorts
   a) complete follow up - all subjects accounted for Ø
   b) subjects lost to follow up unlikely to introduce bias - small number lost - > ____ % (select an adequate %) follow up, or description provided of those lost) Ø
   c) follow up rate < ____% (select an adequate %) and no description of those lost
   d) no statement

Figure S1: Newcastle Ottawa Scale;

Study	Group O COVID-19	Group O Total	Group A COVID-19	Group A Total	OR	95% CI
**Study Design: Case-control study**						
Ad'hiah et al., 2020	324	655	360	655	0.80	[0.65; 1.00]
Anderson et al., 2021	5,521	50,920	4,545	43,417	1.04	[1.00; 1.08]
Negro et al., 2021	69	451	77	426	0.82	[0.57; 1.17]
Platton et al., 2021	8	19	8	16	0.73	[0.19; 2.77]
Singh et al., 2021	13	35	19	36	0.53	[0.20; 1.36]
**All studies**	**5,935**	**52,080**	**5,009**	**44,550**	**0.89**	**[0.73; 1.08]**
Heterogeneity: $I^2 = 56\%$, $\tau^2 = 0.0221$, $p = 0.06$						
Test for effect: $z = -1.16$ ($p = 0.25$)						
**Study Design: Cohort / Cross-sectional study**						
Ahmed et al., 2021	21	133	34	126	0.51	[0.28; 0.93]
Barnkob et al., 2020	2,851	196,252	3,296	202,507	0.89	[0.85; 0.94]
Boudin et al., 2020	553	742	521	674	0.86	[0.67; 1.10]
Coluk et al., 2021	50	69	69	101	1.22	[0.62; 2.40]
Haizler-Cohen et al., 2021	209	1,319	145	955	1.05	[0.84; 1.32]
Horspool et al., 2021	4	30	4	23	0.73	[0.16; 3.30]
Ibrahim et al., 2021	40	306	18	176	1.32	[0.73; 2.38]
Khosroshahi et al., 2021	12	233	21	225	0.53	[0.25; 1.10]
Latz et al., 2020	587	3,656	440	2,649	0.96	[0.84; 1.10]
Lehrer et al., 2021	308	5,390	323	5,516	0.97	[0.83; 1.14]
Levi et al., 2021	913	3,002	816	2,505	0.90	[0.81; 1.01]
Mahallawi et al., 2021	76	503	117	401	0.43	[0.31; 0.60]
Niles et al., 2020	17,739	136,513	11,017	93,750	1.12	[1.09; 1.15]
Quiroga et al., 2021	19	124	34	155	0.64	[0.35; 1.20]
Ray et al., 2021	2,883	100,002	2,420	81,797	0.97	[0.92; 1.03]
Schetelig et al., 2021	1,916	40,975	2,345	44,131	0.87	[0.82; 0.93]
Zhang et al., 2021	682	7,466	728	7,744	0.97	[0.87; 1.08]
**All studies**	**28,863**	**496,715**	**22,348**	**443,435**	**0.91**	**[0.83; 0.99]**
Heterogeneity: $I^2 = 90\%$, $\tau^2 = 0.0191$, $p < 0.01$						
Test for effect: $z = -2.13$ ($p = 0.03$)						
**Random effects model**	**34,798**	**548,795**	**27,357**	**487,985**	**0.91**	**[0.85; 0.98]**
Heterogeneity: $I^2 = 87\%$, $\tau^2 = 0.0144$, $p < 0.01$						
Residual heterogeneity: $I^2 = 88\%$, $p < 0.01$						
Test for overall effect: $z = -2.48$ ($p = 0.01$)						

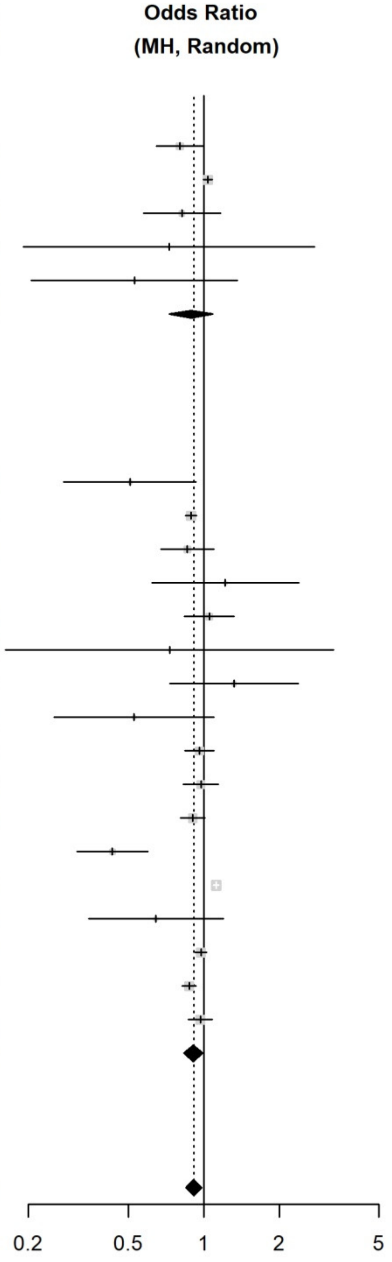

Figure S2: Forest plot for the occurrence of SARS-CoV-2 infection in patients with Blood Group O versus Group A

Study	Group O COVID-19	Group O Total	Group B COVID-19	Group B Total	Odds Ratio (MH, Random)	OR	95% CI
**Study Design: Case-control study**							
Ad'hiah et al., 2020	324	655	221	415		0.86	[0.67; 1.10]
Anderson et al., 2021	5,521	50,920	1,037	9,949		1.05	[0.97; 1.12]
Negro et al., 2021	69	451	15	131		1.40	[0.77; 2.53]
Platton et al., 2021	8	19	3	4		0.24	[0.02; 2.78]
Singh et al., 2021	13	35	13	21		0.36	[0.12; 1.11]
**All studies**	**5,935**	**52,080**	**1,289**	**10,520**		**0.96**	**[0.76; 1.21]**
Heterogeneity: $I^2 = 50\%$, $\tau^2 = 0.0274$, $p = 0.09$							
Test for effect: $z = -0.35$ ($p = 0.72$)							
**Study Design: Cohort / Cross-sectional study**							
Ahmed et al., 2021	21	133	19	67		0.47	[0.23; 0.96]
Barnkob et al., 2020	2,851	196,252	897	53,735		0.87	[0.81; 0.94]
Boudin et al., 2020	553	742	135	183		1.04	[0.72; 1.50]
Coluk et al., 2021	50	69	20	32		1.58	[0.65; 3.84]
Haizler-Cohen et al., 2021	209	1,319	72	508		1.14	[0.85; 1.52]
Horspool et al., 2021	4	30	2	9		0.54	[0.08; 3.57]
Ibrahim et al., 2021	40	306	7	88		1.74	[0.75; 4.03]
Khosroshahi et al., 2021	12	233	11	160		0.74	[0.32; 1.71]
Latz et al., 2020	587	3,656	201	1,035		0.79	[0.66; 0.95]
Lehrer et al., 2021	308	5,390	66	1,238		1.08	[0.82; 1.41]
Levi et al., 2021	913	3,002	237	713		0.88	[0.74; 1.04]
Mahallawi et al., 2021	76	503	32	253		1.23	[0.79; 1.92]
Niles et al., 2020	17,739	136,513	4,282	36,140		1.11	[1.07; 1.15]
Quiroga et al., 2021	19	124	5	33		1.01	[0.35; 2.95]
Ray et al., 2021	2,883	100,002	1,378	33,536		0.69	[0.65; 0.74]
Schetelig et al., 2021	1,916	40,975	570	11,866		0.97	[0.88; 1.07]
Zhang et al., 2021	682	7,466	177	1,763		0.90	[0.76; 1.07]
**All studies**	**28,863**	**496,715**	**8,111**	**141,359**		**0.94**	**[0.82; 1.07]**
Heterogeneity: $I^2 = 91\%$, $\tau^2 = 0.0460$, $p < 0.01$							
Test for effect: $z = -0.95$ ($p = 0.34$)							
**Random effects model**	**34,798**	**548,795**	**9,400**	**151,879**		**0.94**	**[0.84; 1.05]**
Heterogeneity: $I^2 = 89\%$, $\tau^2 = 0.0371$, $p < 0.01$							
Residual heterogeneity: $I^2 = 90\%$, $p < 0.01$							
Test for overall effect: $z = -1.14$ ($p = 0.26$)							

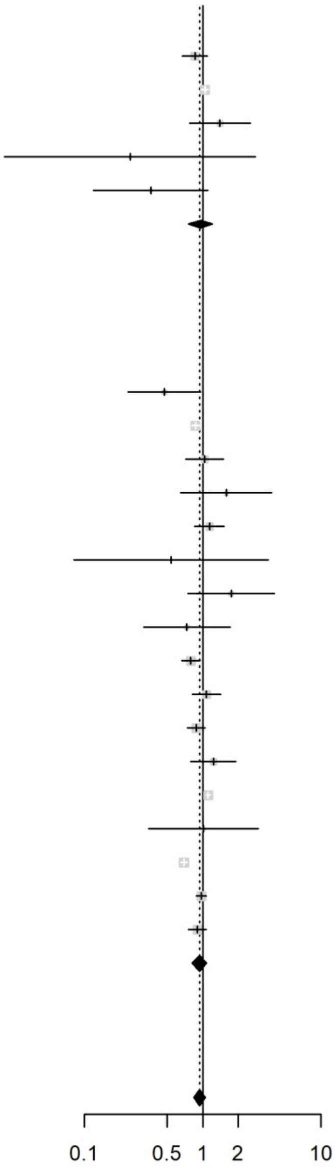

Figure S3: Forest plot for the occurrence of SARS-CoV-2 infection in patients with Blood Group O versus Group B

Study	Group O COVID-19	Group O Total	Group AB COVID-19	Group AB Total	Odds Ratio (MH, Random)	OR	95% CI
**Study Design: Case-control study**							
Ad'hiah et al., 2020	324	655	109	190		0.73	[0.53; 1.01]
Anderson et al., 2021	5,521	50,920	365	3,510		1.05	[0.94; 1.17]
Negro et al., 2021	69	451	6	50		1.32	[0.54; 3.23]
Platton et al., 2021	8	19	1	1		0.25	[0.01; 6.82]
Singh et al., 2021	13	35	5	8		0.35	[0.07; 1.73]
**All studies**	**5,935**	**52,080**	**486**	**3,759**		**0.90**	**[0.67; 1.21]**
Heterogeneity: $I^2 = 43\%$, $\tau^2 = 0.0395$, $p = 0.13$							
Test for effect: $z = -0.67$ ($p = 0.50$)							
**Study Design: Cohort / Cross-sectional study**							
Ahmed et al., 2021	21	133	12	29		0.27	[0.11; 0.64]
Barnkob et al., 2020	2,851	196,252	378	21,160		0.81	[0.73; 0.90]
Boudin et al., 2020	553	742	54	70		0.87	[0.48; 1.55]
Coluk et al., 2021	50	69	5	9		2.11	[0.51; 8.68]
Haizler-Cohen et al., 2021	209	1,319	22	148		1.08	[0.67; 1.74]
Horspool et al., 2021	4	30	0	2		0.85	[0.03; 20.76]
Ibrahim et al., 2021	40	306	1	16		2.26	[0.29; 17.55]
Khosroshahi et al., 2021	12	233	0	52		5.93	[0.35; 101.69]
Latz et al., 2020	587	3,656	61	308		0.77	[0.58; 1.04]
Lehrer et al., 2021	308	5,390	23	431		1.08	[0.70; 1.66]
Levi et al., 2021	913	3,002	71	237		1.02	[0.77; 1.36]
Mahallawi et al., 2021	76	503	9	55		0.91	[0.43; 1.94]
Niles et al., 2020	17,739	136,513	1,153	10,133		1.16	[1.09; 1.24]
Quiroga et al., 2021	19	124	2	8		0.54	[0.10; 2.89]
Ray et al., 2021	2,883	100,002	390	10,221		0.75	[0.67; 0.83]
Schetelig et al., 2021	1,916	40,975	285	5,370		0.88	[0.77; 0.99]
Zhang et al., 2021	682	7,466	56	613		1.00	[0.75; 1.33]
**All studies**	**28,863**	**496,715**	**2,522**	**48,862**		**0.91**	**[0.78; 1.05]**
Heterogeneity: $I^2 = 81\%$, $\tau^2 = 0.0452$, $p < 0.01$							
Test for effect: $z = -1.29$ ($p = 0.20$)							
**Random effects model**	**34,798**	**548,795**	**3,008**	**52,621**		**0.91**	**[0.80; 1.03]**
Heterogeneity: $I^2 = 77\%$, $\tau^2 = 0.0369$, $p < 0.01$							
Residual heterogeneity: $I^2 = 78\%$, $p < 0.01$							
Test for overall effect: $z = -1.54$ ($p = 0.12$)							

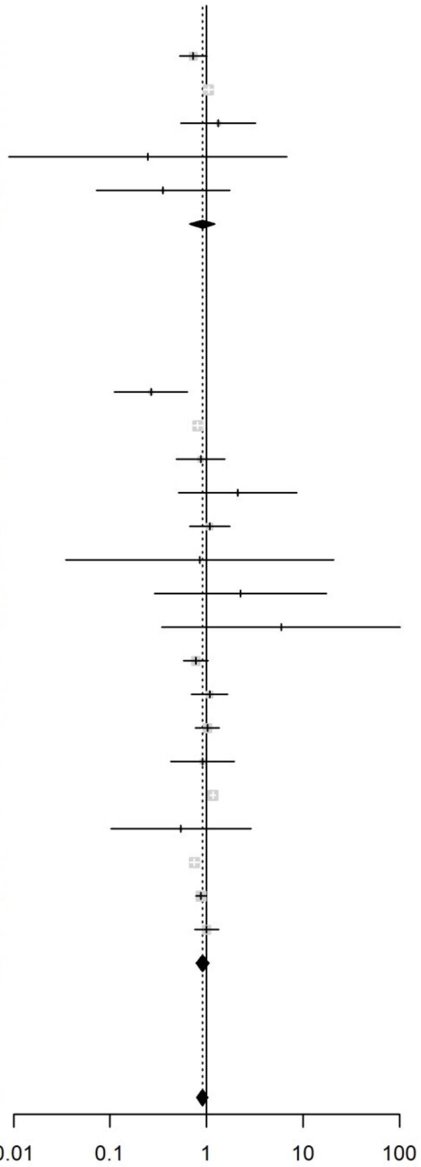

Figure S4: Forest plot for the occurrence of SARS-CoV-2 infection in patients with Blood Group O versus Group AB

Study	Group A COVID-19	Group A Total	Group B COVID-19	Group B Total	OR	95% CI
**Study Design: Case-control study**						
Ad’hiah et al., 2020	360	655	221	415	1.07	[0.84; 1.37]
Anderson et al., 2021	4,545	43,417	1,037	9,949	1.00	[0.94; 1.08]
Negro et al., 2021	77	426	15	131	1.71	[0.94; 3.08]
Platton et al., 2021	8	16	3	4	0.33	[0.03; 3.93]
Singh et al., 2021	19	36	13	21	0.69	[0.23; 2.06]
**All studies**	**5,009**	**44,550**	**1,289**	**10,520**	**1.03**	**[0.92; 1.16]**
Heterogeneity: $I^2 = 11\%$, $\tau^2 = 0.0034$, $p = 0.34$						
Test for effect: $z = 0.50$ ($p = 0.62$)						
**Study Design: Cohort / Cross-sectional study**						
Ahmed et al., 2021	34	126	19	67	0.93	[0.48; 1.81]
Barnkob et al., 2020	3,296	202,507	897	53,735	0.97	[0.90; 1.05]
Boudin et al., 2020	521	674	135	183	1.21	[0.83; 1.76]
Coluk et al., 2021	69	101	20	32	1.29	[0.56; 2.97]
Haizler-Cohen et al., 2021	145	955	72	508	1.08	[0.80; 1.47]
Horspool et al., 2021	4	23	2	9	0.74	[0.11; 4.96]
Ibrahim et al., 2021	18	176	7	88	1.32	[0.53; 3.29]
Khosroshahi et al., 2021	21	225	11	160	1.39	[0.65; 2.98]
Latz et al., 2020	440	2,649	201	1,035	0.83	[0.69; 0.99]
Lehrer et al., 2021	323	5,516	66	1,238	1.10	[0.84; 1.45]
Levi et al., 2021	816	2,505	237	713	0.97	[0.81; 1.16]
Mahallawi et al., 2021	117	401	32	253	2.85	[1.85; 4.37]
Niles et al., 2020	11,017	93,750	4,282	36,140	0.99	[0.95; 1.03]
Quiroga et al., 2021	34	155	5	33	1.57	[0.56; 4.38]
Ray et al., 2021	2,420	81,797	1,378	33,536	0.71	[0.67; 0.76]
Schetelig et al., 2021	2,345	44,131	570	11,866	1.11	[1.01; 1.22]
Zhang et al., 2021	728	7,744	177	1,763	0.93	[0.78; 1.11]
**All studies**	**22,348**	**443,435**	**8,111**	**141,359**	**1.03**	**[0.92; 1.15]**
Heterogeneity: $I^2 = 87\%$, $\tau^2 = 0.0296$, $p < 0.01$						
Test for effect: $z = 0.48$ ($p = 0.63$)						
**Random effects model**	**27,357**	**487,985**	**9,400**	**151,879**	**1.03**	**[0.93; 1.13]**
Heterogeneity: $I^2 = 84\%$, $\tau^2 = 0.0238$, $p < 0.01$						
Residual heterogeneity: $I^2 = 84\%$, $p < 0.01$						
Test for overall effect: $z = 0.56$ ($p = 0.58$)						

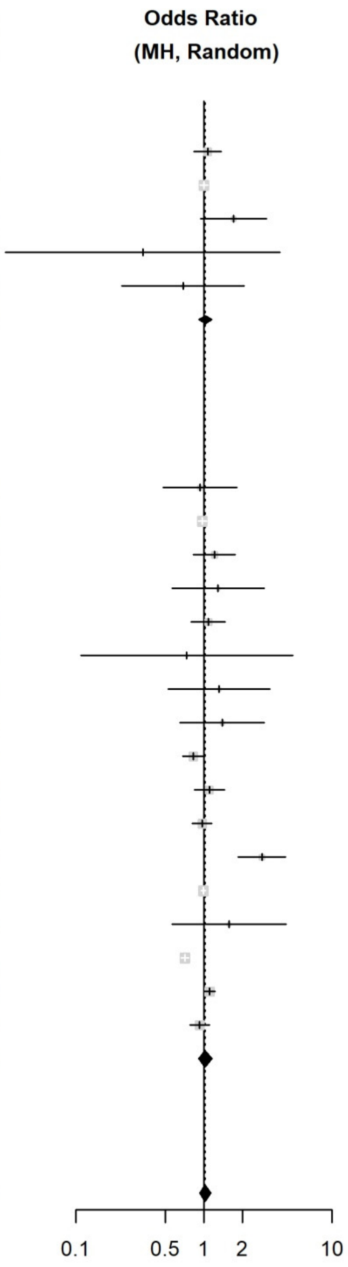

Figure S5: Forest plot for the occurrence of SARS-CoV-2 infection in patients with Blood Group A versus Group B

Study	Group A COVID-19	Group A Total	Group AB COVID-19	Group AB Total	OR	95% CI
**Study Design: Case-control study**						
Ad'hiah et al., 2020	360	655	109	190	0.91	[0.65; 1.26]
Anderson et al., 2021	4,545	43,417	365	3,510	1.01	[0.90; 1.13]
Negro et al., 2021	77	426	6	50	1.62	[0.67; 3.93]
Platton et al., 2021	8	16	1	1	0.33	[0.01; 9.40]
Singh et al., 2021	19	36	5	8	0.67	[0.14; 3.24]
**All studies**	**5,009**	**44,550**	**486**	**3,759**	**1.00**	**[0.90; 1.11]**
Heterogeneity: $I^2 = 0\%$, $\tau^2 = 0$, $p = 0.71$						
Test for effect: $z = 0.00$ ($p = 1.00$)						
**Study Design: Cohort / Cross-sectional study**						
Ahmed et al., 2021	34	126	12	29	0.52	[0.23; 1.21]
Barnkob et al., 2020	3,296	202,507	378	21,160	0.91	[0.82; 1.01]
Boudin et al., 2020	521	674	54	70	1.01	[0.56; 1.81]
Coluk et al., 2021	69	101	5	9	1.73	[0.43; 6.86]
Haizler-Cohen et al., 2021	145	955	22	148	1.03	[0.63; 1.67]
Horspool et al., 2021	4	23	0	2	1.15	[0.05; 28.44]
Ibrahim et al., 2021	18	176	1	16	1.71	[0.21; 13.71]
Khosroshahi et al., 2021	21	225	0	52	11.04	[0.66; 185.24]
Latz et al., 2020	440	2,649	61	308	0.81	[0.60; 1.09]
Lehrer et al., 2021	323	5,516	23	431	1.10	[0.71; 1.70]
Levi et al., 2021	816	2,505	71	237	1.13	[0.85; 1.51]
Mahallawi et al., 2021	117	401	9	55	2.11	[1.00; 4.44]
Niles et al., 2020	11,017	93,750	1,153	10,133	1.04	[0.97; 1.11]
Quiroga et al., 2021	34	155	2	8	0.84	[0.16; 4.37]
Ray et al., 2021	2,420	81,797	390	10,221	0.77	[0.69; 0.86]
Schetelig et al., 2021	2,345	44,131	285	5,370	1.00	[0.88; 1.14]
Zhang et al., 2021	728	7,744	56	613	1.03	[0.78; 1.37]
**All studies**	**22,348**	**443,435**	**2,522**	**48,862**	**0.96**	**[0.87; 1.07]**
Heterogeneity: $I^2 = 56\%$, $\tau^2 = 0.0140$, $p < 0.01$						
Test for effect: $z = -0.73$ ($p = 0.47$)						
**Random effects model**	**27,357**	**487,985**	**3,008**	**52,621**	**0.96**	**[0.89; 1.05]**
Heterogeneity: $I^2 = 46\%$, $\tau^2 = 0.0097$, $p < 0.01$						
Residual heterogeneity: $I^2 = 48\%$, $p < 0.01$						
Test for overall effect: $z = -0.86$ ($p = 0.39$)						

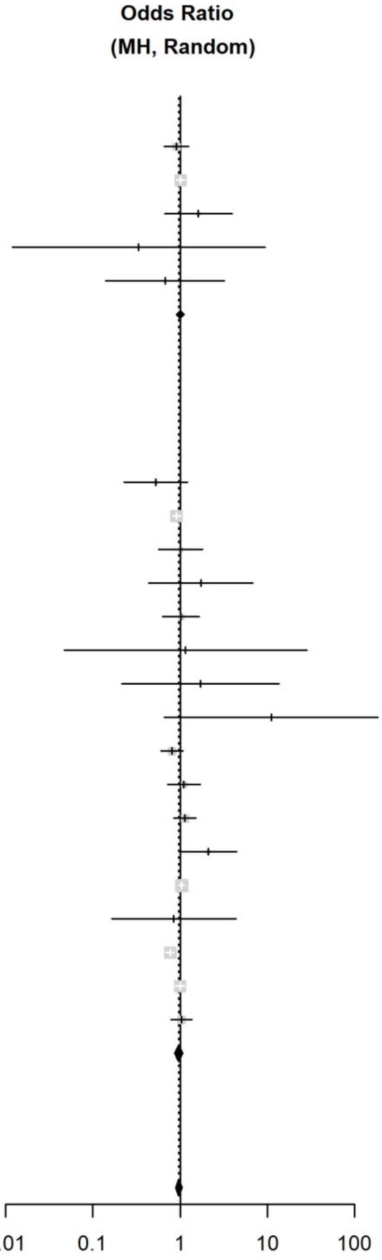

Figure S6: Forest plot for the occurrence of SARS-CoV-2 infection in patients with Blood Group A versus Group AB

Study	Group B COVID-19	Group B Total	Group AB COVID-19	Group AB Total	OR	95% CI
**Study Design: Case-control study**						
Ad'hiah et al., 2020	221	415	109	190	0.85	[0.60; 1.20]
Anderson et al., 2021	1,037	9,949	365	3,510	1.00	[0.88; 1.14]
Negro et al., 2021	15	131	6	50	0.95	[0.35; 2.60]
Platton et al., 2021	3	4	1	1	0.78	[0.02; 32.37]
Singh et al., 2021	13	21	5	8	0.98	[0.18; 5.23]
**All studies**	**1,289**	**10,520**	**486**	**3,759**	**0.98**	**[0.87; 1.10]**
Heterogeneity: I^2 = 0%, τ^2 = 0, p = 0.93						
Test for effect: z = -0.30 (p = 0.76)						
**Study Design: Cohort / Cross-sectional study**						
Ahmed et al., 2021	19	67	12	29	0.56	[0.23; 1.39]
Barnkob et al., 2020	897	53,735	378	21,160	0.93	[0.83; 1.05]
Boudin et al., 2020	135	183	54	70	0.83	[0.44; 1.59]
Coluk et al., 2021	20	32	5	9	1.33	[0.30; 5.96]
Haizler-Cohen et al., 2021	72	508	22	148	0.95	[0.56; 1.59]
Horspool et al., 2021	2	9	0	2	1.67	[0.06; 47.83]
Ibrahim et al., 2021	7	88	1	16	1.30	[0.15; 11.31]
Khosroshahi et al., 2021	11	160	0	52	8.08	[0.47; 139.47]
Latz et al., 2020	201	1,035	61	308	0.98	[0.71; 1.34]
Lehrer et al., 2021	66	1,238	23	431	1.00	[0.61; 1.63]
Levi et al., 2021	237	713	71	237	1.16	[0.85; 1.60]
Mahallawi et al., 2021	32	253	9	55	0.74	[0.33; 1.66]
Niles et al., 2020	4,282	36,140	1,153	10,133	1.05	[0.98; 1.12]
Quiroga et al., 2021	5	33	2	8	0.54	[0.08; 3.45]
Ray et al., 2021	1,378	33,536	390	10,221	1.08	[0.96; 1.21]
Schetelig et al., 2021	570	11,866	285	5,370	0.90	[0.78; 1.04]
Zhang et al., 2021	177	1,763	56	613	1.11	[0.81; 1.52]
**All studies**	**8,111**	**141,359**	**2,522**	**48,862**	**1.02**	**[0.97; 1.06]**
Heterogeneity: I^2 = 0%, τ^2 = 0, p = 0.69						
Test for effect: z = 0.62 (p = 0.54)						
**Random effects model**	**9,400**	**151,879**	**3,008**	**52,621**	**1.01**	**[0.97; 1.06]**
Heterogeneity: I^2 = 0%, τ^2 = 0, p = 0.87						
Residual heterogeneity: I^2 = 0%, p = 0.85						
Test for overall effect: z = 0.46 (p = 0.65)						

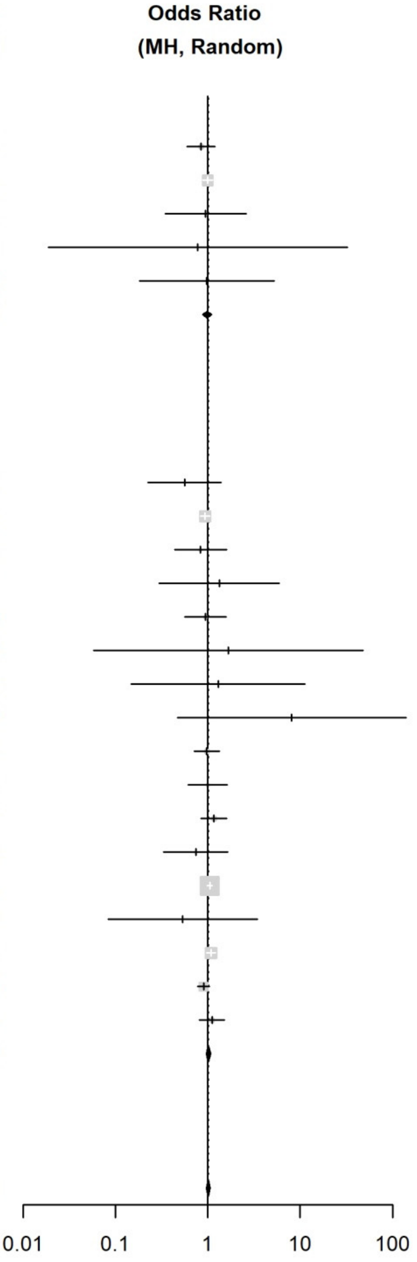

Figure S7: Forest plot for the occurrence of SARS-CoV-2 infection in patients with Blood Group B versus Group AB.
